# Comparative risk assessment for the development of cardiovascular diseases in the Hungarian general and Roma population

**DOI:** 10.1038/s41598-021-82689-0

**Published:** 2021-02-04

**Authors:** Peter Piko, Zsigmond Kosa, Janos Sandor, Roza Adany

**Affiliations:** 1 MTA-DE Public Health Research Group, Public Health Research Institute, University of Debrecen, Debrecen, 4028 Hungary; 2 Department of Health Visitor Methodology and Public Health, Faculty of Health, University of Debrecen, Nyíregyháza, 4400 Hungary; 3 Department of Public Health and Epidemiology, Faculty of Medicine, University of Debrecen, Debrecen, 4028 Hungary

**Keywords:** Cardiology, Population screening

## Abstract

Cardiovascular diseases (CVDs) are the number one cause of death globally, and the early identification of high risk is crucial to prevent the disease and to reduce healthcare costs. Short life expectancy and increased mortality among the Roma are generally accepted (although not indeed proven by mortality analyses) which can be partially explained by the high prevalence of cardiovascular risk factors (CVRF) among them. This study aims to elaborate on the prevalence of the most important CVD risk factors, assess the estimation of a 10-year risk of development of fatal and nonfatal CVDs based on the most used risk assessment scoring models, and to compare the Hungarian general (HG) and Roma (HR) populations. In 2018 a complex health survey was accomplished on the HG (n = 380) and HR (n = 347) populations. The prevalence of CVRS was defined and 10-year cardiovascular risk was estimated for both study populations using the following systems: Framingham Risk Score for hard coronary heart disease (FRS_CHD_) and for cardiovascular disease (FRS_CVD_), Systematic COronary Risk Evaluation (SCORE), ACC/AHA Pooled Cohort Equations (PCE) and Revised Pooled Cohort Equations (RPCE). After the risk scores had been calculated, the populations were divided into risk categories and all subjects were classified. For all CVD risk estimation scores, the average of the estimated risk was higher among Roma compared to the HG independently of the gender. The proportion of high-risk group in the Hungarian Roma males population was on average 1.5–3 times higher than in the general one. Among Roma females, the average risk value was higher than in the HG one. The proportion of high-risk group in the Hungarian Roma females population was on average 2–3 times higher compared to the distribution of females in the general population. Our results show that both genders in the Hungarian Roma population have a significantly higher risk for a 10-year development of cardiovascular diseases and dying from them compared to the HG one. Therefore, cardiovascular interventions should be focusing not only on reducing smoking among Roma but on improving health literacy and service provision regarding prevention, early recognition, and treatment of lipid disorders and diabetes among them.

## Introduction

Of the 55.9 million deaths worldwide in 2017, non-communicable diseases (NCDs) accounted for 73.4%. Among NCDs, the largest number of deaths (17.8 million worldwide) were estimated for cardiovascular diseases (CVDs)^1^. Each year CVDs (mainly ischaemic heart disease and stroke) cause about 3.9 million deaths in Europe, over 1.8 million deaths in the European Union (EU). Although the CVD burden showed a steady decrease during the last few decades in the EU countries, a severe East–West gap in mortality still exists. Central and Eastern Europe (CEE) including the eleven post-communist countries joining the EU since 2004 is the region with the highest CVD burden in world^2^. The standardized death rate for CVDs—the leading cause of death in the country—was 588.15 per 100,000 inhabitants in 2017 in Hungary (5th worst among the EU-28 countries), 1.6 times higher than the EU average (369.46 deaths per 100,000)^1^. Life expectancy increased between 2000 and 2017 (from 71.9 to 76.0 years) in Hungary; a phenomenon that can be explained primarily by the decrease in deaths due to cardiovascular diseases, and especially to stroke. Although the IHD-related death rate decreased by 12% in Hungary between 2000 and 2016, it is still significantly lower than the EU average decrease of more than 40%.

It is reasonable to suppose that the unfavourable CVD mortality figures are at least partly linked with the high representation of the Roma population in the majority of CEE countries^3^. With an estimated number of 10–12 million people, the Roma population is the largest ethnic group in Europe. They live concentrated in the countries of the CEE region (in Bulgaria, Hungary, Slovakia, and Romania)^4^ and their representation in Hungary is about 8–10% of the total population^5,6^. They are concentrated in the Southwest and the Northeast regions of the country, where they frequently live in segregated colonies with severe environmental problems, such as the lack of sewage and gas mains, garbage deposits, waterlogged soil, and lack of water mains^7^. In addition to segregation and deprived living conditions low educational attainment and labour market barriers also measurably exist^8^. These factors combined adversely affect their health indicators. Estimating the scale of the problem is further encumbered by the fact that the collection of data related to health and health-care utilization on an ethnic basis is difficult in countries where Roma live^9^. One-off surveys indicate that they suffer from poor health and have only limited access to healthcare^10–12^.

Increased mortality and short life expectancy of the Roma is not proven (the ethnicity is not recorded in the mortality statistics), but suspected on the basis of the high prevalence of health risk factors among them. Several studies have examined the prevalence of cardiovascular risk factors among the Roma minority and compared it with that of the general population^13–15^. Obesity (France^16^, Hungary^17^, Romania^15^, Slovakia^18–20^, Spain^21^), diabetes (France^16^, Hungary^17^, Serbia^22^, Slovakia^19^), insulin resistance (Slovakia^20^), smoking (Croatia^23^, France^16^, Romania^15,24^, Slovakia^20,25^), physical inactivity (Romania^26^, Slovakia^27^), hypertension (France^16^, Hungary^17^, Slovakia^19^), abnormal lipid profile (Hungary^17^, Romania^15^, Slovakia^19^), and metabolic syndrome (Hungary^17^, Slovakia^14,19^) have high prevalence and were significantly more common among the Roma, regardless of the country in which they live. Roma CHD patients have a worse risk profile at the entry of care and seem to be undertreated compared with non-Roma CHD patients^28^.

The high representation of CVD risk factors can be explained by the impact of environmental factors above mentioned and also by genetic causes (such as in the case of type 2 diabetes mellitus^29^). Based on our previous studies, we can state that the higher risk/prevalence of the venous thrombosis^30^ and reduced high-density lipoprotein cholesterol levels^31–33^ are determined by genetic factors, whereas the high risk/prevalence of type 2 diabetes^34^ and obesity^35^ are influenced by environmental factors in the Hungarian Roma population. Although several studies have examined the prevalence of CVD risk factors in the Roma population, currently, there are no studies focusing on their combined effect in CVD morbidity or mortality risk prediction models.

About 360 predictive estimation models have been identified by Damen et al.^36^ that might be used to develop targeted primary prevention and intervention against the development of CVDs. The most widely used tools for estimating cardiovascular risk in a population are the Framingham Risk Score (FRS^37^), the Systematic COronary Risk Evaluation (SCORE^38^), the Pooled Cohort Equations (PCE^39^) and Revised Pooled Cohort Equations (RPCE^40^) introduced by American College of Cardiology (ACC) and American Heart Association (AHA). Currently, the most widely praised clinical practice guidelines (Canadian Cardiovascular Society^41^, European Society of Cardiology/European Society of Hypertension^42^, ACC/AHA^43^, Joint British Societies recommendations on the prevention of Cardiovascular Disease^44^) are used to estimate the future risk of CVD applying the total/global/absolute risk score for CVDs. Over the past two decades, several randomised control trials have examined the effectiveness of CVD risk scores^45–47^. These charts include traditional cardiovascular risk factors, such as age, gender, smoking status, presence of diabetes, blood pressure, high-density lipoprotein- (HDL-C), and total cholesterol (TC) levels.

The present study was conducted in order to compare the 10-year cardiovascular risk prediction scores in the Hungarian general and Roma populations and to identify high-risk individuals who might be targeted by cost-effective cardiovascular interventions. In order to ensure the comparability of our data with findings obtained in other studies, we used the three most common risk estimation models/methods (FRS, SCORE, and ACC/AHA PCE) and their revised version for our study.

## Material and methods

### Study populations

#### Sample representative of the Hungarian Roma (HR) population living in segregated colonies in Northeast Hungary

The Hungarian Roma sample population was enrolled from Hajdú-Bihar and Szabolcs-Szatmár-Bereg counties in Northeast Hungary. The majority of the segregated Hungarian Roma population lives in these two counties. There were 92 segregated colonies identified and 25 of them were selected randomly using general practitioners' (GPs) validated household lists. Twenty individuals (one person per household between the ages of 20 and 64) were randomly chosen from each segregated colony and each of them was interviewed face-to-face by Roma ethnic university students under the supervision of a public health coordinator. In addition to this, they were invited to visit a GP for a physical examination and blood collection. The ethnicity of the study participants was identified based on self-declaration.

#### Sample representative of the Hungarian general (HG) population living in Northeast Hungary

The Hungarian general sample population consisted of people randomly selected from the same counties as the Hungarian Roma one. These people are between 20 and 64 years of age and were registered by GPs involved in the General Practitioners’ Morbidity Sentinel Stations Programme (GPMSSP). The GPMSSP was established in 1998 to monitor the incidence and prevalence of chronic non-communicable diseases of major public health importance^48^. Twenty GPs were randomly chosen and 25 individuals from each GP were included in the study. If a person could not be reached, a new one was included instead, but is someone refused to respond, no substitute person could be drawn. Further details on the study design and sample populations can be read in our previous research paper^49^. Briefly, based on the results obtained in a complex three pillars (questionnaire, physical and laboratory examinations) health (interview and examination) survey a database was created, which—among others—consists all the data necessary the CVD risk calculations in the models we used.

Regarding both study populations known pregnancy was an exclusion criterion during sample collection. Individuals diagnosed with any form of cardiovascular diseases (coronary heart disease, cerebrovascular disease, peripheral arterial disease, rheumatic heart disease, congenital heart disease, deep vein thrombosis and pulmonary embolism) were also excluded from the CVD risk score calculations.

### Ethics declarations

All procedures performed in studies involving human participants were carried out by the ethical standards of the institutional and national research committee and with the 1964 Helsinki declaration and its later amendments. This study was approved by the Ethical Committee of the University of Debrecen, Medical Health Sciences Centre (reference No. 2462-2006), and by the Ethics Committee of the Hungarian Scientific Council on Health (reference No. 61327-2017/EKU). All participants provided written informed consent to participate in the study. This article does not contain any studies with animals performed by any of the authors.

#### Cardiovascular risk stratification models in our study

In the present study, we estimate the cardiovascular risk of Hungarian general and Roma populations in Northeast Hungary using the three most commonly used risk estimation models (FRS, SCORE, PCE) and their revised version. For more detailed characteristics of the cardiovascular risk assessment models used in the study see Table 1.

**Table 1:** Characteristics of cardiovascular risk assessment models used in the study.

	Framingham Risk Scores (FRSs)		Systematic COronary Risk Evaluation (SCORE)	American College of Cardiology/American Heart Association Pooled Cohort Equations	
Characteristics	ATPIII hard coronary heart disease (FRS_CHD_)	Cardiovascular diseases generally (FRS_CVD_)	High-risk algorithm	Original (PCE)	Revised (RPCE)
Year of development	2002	2008	2003	2013	2018
Age group for which applicable (years)	30–75	30–75	40–65	40–79	40–79
Populations for which was validated	Predominantly individuals of Western European descent living in United States of America		European countries with high CVD risk (e.g.: Hungary)	Multiethnic cohorts living in United States of America	
Parameters included	Age, gender, total cholesterol, HDL-cholesterol, systolic blood pressure, BP treatment, and smoking status	Age, gender, total cholesterol, HDL-cholesterol, systolic blood pressure, BP treatment, diabetes, and smoking status	Age, gender, total cholesterol, HDL-cholesterol, systolic blood pressure, smoking status	Age, gender, race, total cholesterol, HDL-cholesterol, systolic blood pressure, BP treatment, diabetes, and smoking status	
Parameters excluded	Diabetes status, family history of CVD	Family history of CVD	BP treatment, diabetes status, family history of CVD	Family history of CVD	
Endpoints	CHD death and nonfatal MI	CHD death, nonfatal myocardial infarction, coronary insufficiency or angina, fatal or nonfatal stroke, and heart failure	Fatal atherosclerotic CVD events (including CHD, arrhythmia, heart failure, stroke, aortic aneurysm, and peripheral vascular disease)	CHD death, nonfatal MI, fatal stroke, nonfatal stroke	

#### Framingham risk score for ATPIII hard coronary heart disease (FRS_CHD_) and for cardiovascular disease generally (FRS_CVD_)

The Framingham Risk Score calculation as a gender-specific algorithm was first developed to assess the 10-year risk of the development of coronary heart disease (CHD) for individuals with different combinations of risk factors based on data obtained from the Framingham Heart Study^50^. The Framingham Risk Score was modified (in 2002) by the third Adult Treatment Panel (ATP III) by the elimination of diabetes from the algorithm since it was considered to be a coronary heart disease (CHD) equivalent; broadening of the age range, by the inclusion of hypertension treatment, age-specific points for smoking and total cholesterol^51^. In our study, the ATPIII based Framingham Risk Score to estimate the 10-year risk of hard coronary heart disease was used.

The original 1998 and revised 2002 Framingham Risk Scores do not include all of the potential manifestations and adverse consequences of atherosclerosis, such as stroke, transient ischemic attack, claudication, and heart failure (although manifestations of aortic atherosclerosis were omitted). These patient-important vascular outcomes were included in the development of the 2008 Framingham general cardiovascular disease risk score, which was shown to have a reliable predictive ability^37^. The estimated risk of developing a cardiovascular event was higher when this risk score was used than in the case of those that predict only CHD events.

The analyses were performed for participants of the study populations aged 30–64, which were divided into the following 10-year risk categories: low (< 10%), intermediate (10–20%), or high (≥ 20%) categories.

#### Systematic COronary Risk Evaluation (SCORE)

SCORE, recommended in the 2007 European Society of Cardiology guidelines on cardiovascular disease prevention in clinical practice, included data on more than 205,178 patients pooled from cohort studies in 12 European countries, 3 million person-years of observation and 7,934 fatal CV events^38,52^. The SCORE model has been calibrated according to each European country’s mortality statistics. A unique aspect of SCORE is that separate risk scores were calculated for high- and low-risk regions of Europe. The predictive value of SCORE was high in each study cohort. Hungary is a high-risk country, and there is no separate data available for the Roma population, so we used the high-risk formula for both populations in our calculations.

SCORE differs from the earlier Framingham risk models (and others) in two important ways: it estimates the 10-year risk of any first fatal atherosclerotic event (e.g. stroke or ruptured abdominal aneurysm), not only CHD-related deaths, and it estimates CVD mortality. SCORE was counted by an online calculator tool^53^.

The analyses were performed for study populations aged 40–64, which were divided into the following categories: low (< 2%), intermediate (2–5%), or high (≥ 5%) SCORE risk category.

#### American College of Cardiology/American Heart Association Pooled Cohort Equations (PCE) and Revised Pooled Cohort Equations (RPCE)

For decades, FRS was the most widely used to estimate 10-year CVD risk in asymptomatic individuals. Nevertheless, FRS underestimates lifetime risk, especially in younger individuals with multiple risks and in women; moreover, it does not predict the risk of stroke^54,55^, which has made the introduction of the ACC/AHA Pooled Cohort Risk Equations necessary in order to overcome these limitations. In this system, diabetes mellitus was included as a predictor variable, and fatal and nonfatal stroke was added to the CVD endpoints^39,56^.

The 2013 American College of Cardiology/American Heart Association (ACC/AHA) pooled cohort equations model was developed from several United States cohorts and it includes different calculators for Caucasian and African American populations^43^. Outcomes are limited to both fatal and nonfatal CHD and stroke. From 2013, the PCE risk score has been recommended for use in the United States^39,43^.

Several cohorts of patients were used to develop the 2013 ACC/AHA cardiovascular risk calculator^57^, the first risk model which includes data from large populations of both Caucasian and African American patients. The model is based on the same parameters as the 2008 Framingham general CVD model, but in contrast to the 2008 Framingham model, it includes only hard endpoints (fatal and nonfatal MI and stroke). However, while the calculator appears to be well-calibrated in populations similar to those for which the calculator was developed, it has not been that accurate in other populations^58^.

A potential limitation of the ACC/AHA calculator is that a family history of premature CVD is not included in the model. This may result in underestimation of risk in patients with very strong family histories of cardiovascular events. Additionally, the ACC/AHA includes diabetes only as a yes/no choice. Issues that may affect the risk of diabetes include patient age, sex, other cardiovascular risk factors, duration of diabetes, and whether the patient has type 1 or type 2 diabetes mellitus are not considered.

The 2013 PCE for predicting 10-year atherosclerotic CVD risk has been criticized for overestimating risk. Outdated population data and statistical methods were considered as the main weaknesses of PCE. In 2018, a modified version was released that was described using other statistical methods and new populations. This Revised Pooled Cohort Equations model estimates the 10-year risk for atherosclerotic cardiovascular disease which is defined as coronary death or nonfatal myocardial infarction, or fatal or nonfatal stroke^40^.

The analyses we have performed for study populations aged 40–64, which were divided into the following categories: low (< 5%), borderline (5–7.49%), intermediate (7.5–19.99%), or high (≥ 20%) risk category for both risk models.

### Statistical analyses

All statistical tests were conducted using IBM SPSS version 26 (IBM Company, Armonk, NY, USA) software. Mann–Whitney U tests were used to compare the age, systolic, and diastolic blood pressure, total cholesterol and, HDL-C level of the study populations. Mean and 95% confidence interval (95% CI) were used to describe continuous variables. Frequencies of categorical variables were statistically compared by using the chi-squared test. Generally, the conventional p threshold of 0.05 was used.

The following risk estimation models were calculated based on their formula using Microsoft Excel 2013 programme: FRS_CHD_, FRS_CVD_, PCE, and RPCE. For SCORE, the HeartScore online calculator was used^53^. The age group corresponding to the risk calculation model was selected specifically for each study population. Individuals diagnosed with any form of cardiovascular diseases (coronary heart disease, cerebrovascular disease, peripheral arterial disease, rheumatic heart disease, congenital heart disease, deep vein thrombosis and pulmonary embolism) were also excluded from the CVD risk score calculations. To avoid distorting effects the study populations were matched for age.

## Results

### Process of sample selection and prevalence of cardiovascular diseases in the Hungarian general and Hungarian Roma populations

**Figure 1:**
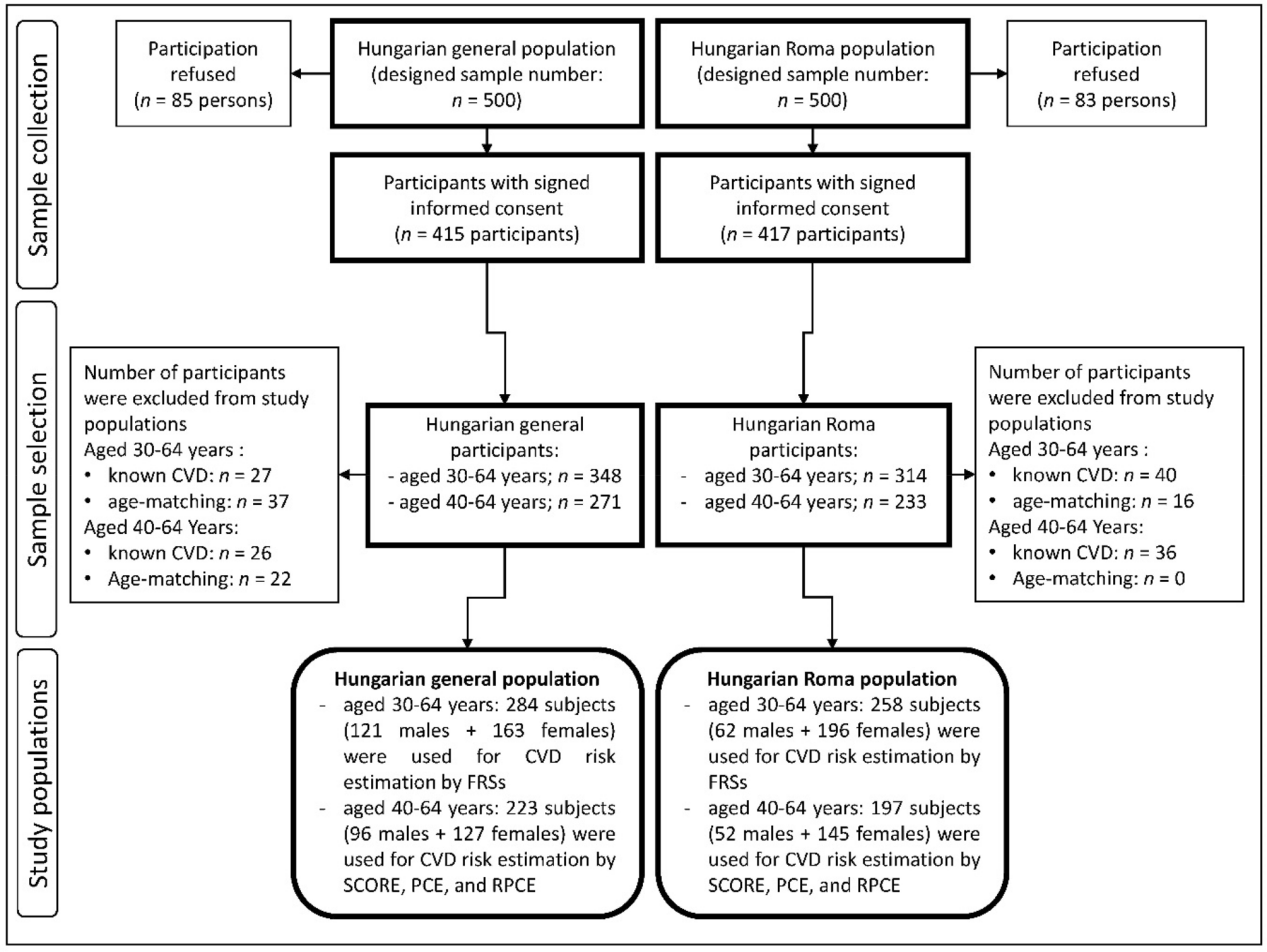
Flowchart showing the process of sample selection for study populations.

The prevalence of cardiovascular diseases was significantly higher in the HR population compared with the HG one (HR: 12.74% vs HG: 7.76%, p = 0.034) in the age group of 30–64 years. Significant difference (p = 0.046) was also observed in the 40–64-year-old age group, 18.27% had CVD in the Hungarian Roma and 10.61% in the Hungarian general population. After exclusion of individuals with any form of CVD (27 persons in the 30–64 age group and 26 persons in the 40–64 age group from the HG population; 40 persons in the 30–64 age group and 36 persons in the 40–64 age group from the HR one) during the process of age matching, 16 h and 37 HG individuals were excluded from the 30–64 age group, while 22 HG were excluded from the 40–64 age group. For more details on the process of sample selection see Fig. 1.

### Characteristics of study populations by age groups

#### Characteristics of 30–64-year-old age group

The characteristics of 30–64-year-old male populations significantly differed in the prevalence of smoking (HG: 35.54% vs. HR: 58.06%, p = 0.004, while female populations showed a significant difference in HDL-C levels (HG: 1.47 vs. HR: 1.29, p < 0.001), the prevalence of smoking (HG: 33.74% vs. HR: 68.37%, p < 0.001), and the prevalence of diabetes (HG: 8.59% vs. HR: 16.33%, p = 0.029). The prevalence of lipid-lowering therapy, indicating indirectly an increased risk of CVD, was higher in both sexes in the Roma population (HR: 12.90% vs. HG: 4.96%, p = 0.056 in males; HR: 11.22% vs. HG: 4.91%, p = 0.031 in females). See more detailed population characteristics in Table 2.

**Table 2:** Characteristics of 30–64-year-old study populations involved in the Framingham Risk Score for hard coronary heart disease (FRS_CHD_) and Framingham Risk Score for cardiovascular disease (FRS_CVD_) calculations by ethnicity and sex.

	Males			Females		
	Hungarian general (n = 121)	Hungarian Roma (n = 62)	p-value	Hungarian general (n = 163)	Hungarian Roma (n = 196)	p-value
	Mean (95% CI)			Mean (95% CI)		
Age (years)	47.98 (46.35–49.60)	49.60 (47.11–52.08)	0.244	47.31 (45.85–48.76)	46.29 (45.03–47.55)	0.274
Total cholesterol levels (mmol/L)	5.13 (4.96–5.31)	5.05 (4.79–5.32)	0.563	5.11 (4.94–5.28)	5.13 (4.97–5.28)	0.931
HDL-C levels (mmol/L)	1.31 (1.24–1.38)	1.23 (1.14–1.33)	0.211	**1.47 (1.41–1.53)**	**1.29 (1.24–1.34)**	**< 0.001**
Systolic blood pressure (mmHg)	130.76 (128.43–133.08)	130.98 (126.24–135.73)	0.370	124.86 (122.65–127.07)	124.94 (122.49–127.40)	0.479

	Prevalence (%)		p-value	Prevalence (%)		p-value
Smokers	**35.54 (27.43–44.33)**	**58.06 (45.65–69.75)**	**0.004**	**33.74 (26.82–41.24)**	**68.37 (61.62–74.57)**	**< 0.001**
Treated for high blood pressure	25.62 (18.48–33.91)	37.10 (25.88–49.49)	0.107	31.90 (25.11–39.33)	35.20 (28.77–42.07)	0.510
Diabetes^a^	10.74 (6.16–17.18)	9.68 (4.14–18.86)	0.823	**8.59 (5.01–13.62)**	**16.33 (11.66–21.98)**	**0.029**
Receiving lipid-lowering therapy^b^	4.96% (2.10–9.93)	12.90% (6.29–22.87)	0.056	**4.91 (2.35**–**9.05)**	**11.22 (7.38**–**16.21)**	**0.031**

Significant differences in mean or prevalence rates are highlighted in bold.

^a^Used only in the FRS_CVD_ calculation.

^b^Not used for risk calculation in any risk assessment models.

#### Characteristics of 40–64-year-old age group

The characteristics of 40–64-year-old male populations of the HG and HR samples significantly differed in the prevalence of smoking (HG: 35.42% vs. HR: 57.69%, p = 0.009), while the female populations in HDL-C levels (HG: 1.48 vs. HR: 1.31, p < 0.001) and the prevalence of smoking (HG: 36.22% vs. HR: 65.52%, p < 0.001). The prevalence of lipid-lowering therapy, indicating indirectly an increased risk of CVD, was higher in both sexes in the Roma population (HR: 15.38% vs. HG: 6.25%, p = 0.069 in males; HR: 14.48% vs. HG: 6.30%, p = 0.029 in females). See more detailed population characteristics in Table 3.

**Table 3:** Characteristics of 40–64-year-old study populations involved in the calculation of SCORE, PCE and, RPCE by ethnicity and sex.

	Males			Females		
	Hungarian general (n = 96)	Hungarian Roma (n = 52)	p-value	Hungarian general (n = 127)	Hungarian Roma (n = 145)	p-value
	Mean (95% CI)			Mean (95% CI)		
Age (years)	51.36 (50.02–52.71)	52.69 (50.69–54.70)	0.292	51.02 (49.81–52.24)	50.24 (49.14–51.35)	0.354
Total cholesterol levels (mmol/L)	5.21 (5.00–5.41)	5.12 (4.82–5.41)	0.476	5.22 (5.04–5.40)	5.31 (5.13–5.50)	0.613
HDL-C levels (mmol/L)	1.33 (1.25–1.41)	1.25 (1.14–1.35)	0.212	**1.48 (1.41–1.55)**	**1.31 (1.25–1.37)**	**< 0.001**
Systolic blood pressure (mmHg)	131.06 (128.51–133.61)	133.08 (127.19–138.52)	0.981	127.19 (124.69–129.70)	128.73 (125.88–131.58)	0.967

	Prevalence (%)		p-value	Prevalence (%)		p-value
Smokers	**35.42 (26.40–45.30)**	**57.69 (44.15–70.40)**	**0.009**	**36.22 (28.25–44.81)**	**65.52 (57.53–72.89)**	**< 0.001**
Treated for high blood pressure^a^	30.21 (21.71–39.88)	48.46 (26.15–52.02)	0.308	37.01 (28.98–45.62)	40.69 (32.94–48.80)	0.534
Diabetes^a^	12.50 (7.02–20.20)	11.54 (4.96–22.24)	0.864	11.02 (6.46–17.34)	18.62 (12.94–25.54)	0.081
Receiving lipid-lowering therapy^b^	6.25 (2.65–12.43)	15.38 (7.55–26.94)	0.069	**6.30 (3.02**–**11.53)**	**14.48 (9.48**–**20.89)**	**0.029**

Significant differences in mean or prevalence rates are highlighted in bold.

^a^Not used for SCORE chart calculation.

^b^Not used for risk calculation in any risk assessment models.

### Comparison of estimated 10-year risk based on Framingham Risk Score for ATPIII hard coronary heart disease (FRS_CHD_) and for cardiovascular disease (FRS_CVD_) between Hungarian general and Roma populations

The average 10-year risk of CHD based on the calculated FRS_CHD_ shows a significant difference between the Hungarian general and Roma males (HG: 7.23% vs. HR: 9.42%, p = 0.023) and females (HG: 1.79% vs. HR: 2.85%, p < 0.001; respectively) populations. In general, the average 10-year risk of CVD is significantly different based on the FRS_CVD_ between the two observed female populations (HG: 6.38% vs. HR: 8.43%, p = 0.035) but not between the males (HG: 13.29% vs. HR: 16.88%, p = 0.075). See more details in Table 4.

**Table 4:** Results obtained by Framingham Risk Score for hard coronary heart disease (FRS_CHD_) and Framingham Risk Score for cardiovascular disease (FRS_CVD_) calculations in the 30–64-year-old Hungarian general and Hungarian Roma populations.

	Males			Females		
	Hungarian general (n = 121)	Hungarian Roma (n = 62)	p-value	Hungarian general (n = 163)	Hungarian Roma (n = 196)	p-value
	Mean (95% CI)			Mean (95% CI)		
FRS_CHD_	**7.23 (6.20–8.25)**	**9.84 (8.00–11.68)**	**0.023**	**1.79 (1.43–2.16)**	**2.85 (2.38–3.31)**	**< 0.001**
FRS_CVD_	13.29 (11.45–15.13)	16.88 (13.52–20.24)	0.075	**6.38 (5.57**–**7.18)**	**8.43 (7.27–9.60)**	**0.035**

Significant differences in mean or prevalence rates are highlighted in bold.

**Figure 2:**
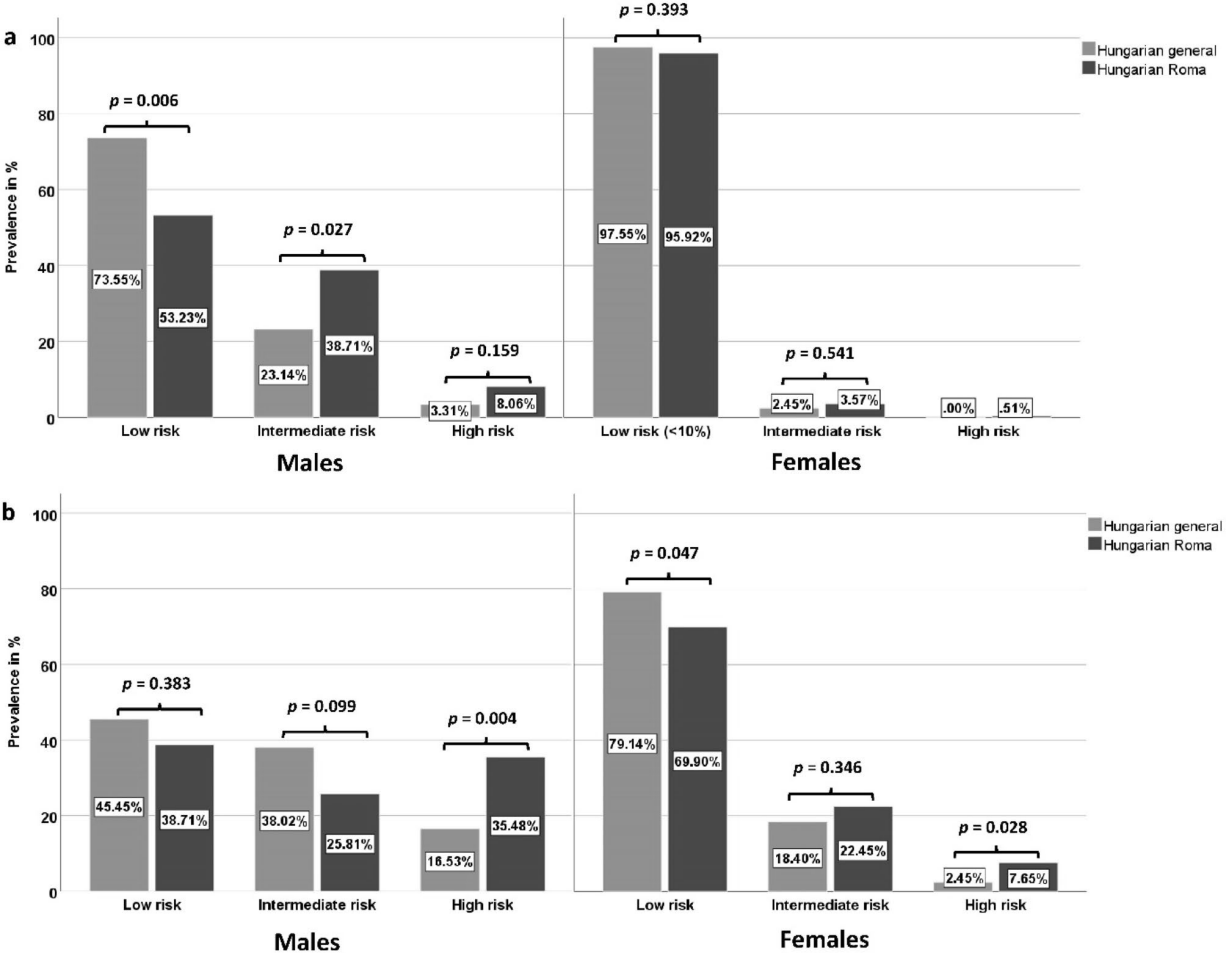
Distribution of subjects by risk categories defined by Framingham Risk Score for hard coronary heart disease (FRSCHD-a) and Framingham Risk Score for cardiovascular disease (FRSCVD-b) calculations in the 30–64-year-old Hungarian general and Hungarian Roma populations.

The relative frequency of low-, intermediate-, and high-risk subjects in the studied male populations differed significantly in cases of both Framingham Risk Score calculations (FRS_CHD_: p = 0.026; FRS_CVD:_ p = 0.020). In case of FRS_CHD,_ the representation of low-risk individuals was significantly lower (HG: 73.55% vs. HR: 53.23%, p = 0.006), while the intermediate- (HG: 23.14% vs. HR: 38.71%, p = 0.027) and high-risk (HG: 3.31% vs. HR: 8.06%, p = 0.159) subjects were more frequent in the Hungarian Roma male population than in the Hungarian general. In case of the FRS_CVD_, people with low- and intermediate cardiovascular risk were more frequent in the Hungarian general male population, while the prevalence of high-risk individuals was 2.15 times higher in the Roma population (HG: 16.53% vs. 35.48%, p = 0.004). The relative frequency of low-, intermediate-, and high-risk subjects in the studied female populations differed significantly in cases of the FRS_CVD_ (p = 0.017) but not in the FRS_CHD_ (p = 0.500), for more details see Fig. 2.

### Comparison of estimated 10-year risk based on Systematic COronary Risk Evaluation (SCORE) between Hungarian general and Roma populations

The average 10-year risk based on SCORE calculated by the HearthScore online tool is significantly different between Hungarian general and Hungarian Roma male populations (HG: 3.83% vs. HR: 5.35%, p = 0.036). No significant difference in risk was detected between the female populations (see more details in Table 5).

**Table 5:** Results of SCORE in the 40–64-year-old Hungarian general and Hungarian Roma populations.

	Males			Females		
	Hungarian general (n = 96)	Hungarian Roma (n = 52)	p-value	Hungarian general (n = 127)	Hungarian Roma (n = 145)	p-value
	Mean (95% CI)			Mean (95% CI)		
SCORE	**3.83 (2.22–4.45)**	**5.35 (3.97–6.72)**	**0.036**	1.28 (1.00–1.55)	1.88 (1.50–2.25)	0.098

Significant differences in mean or prevalence rates are highlighted in bold.

**Figure 3:**
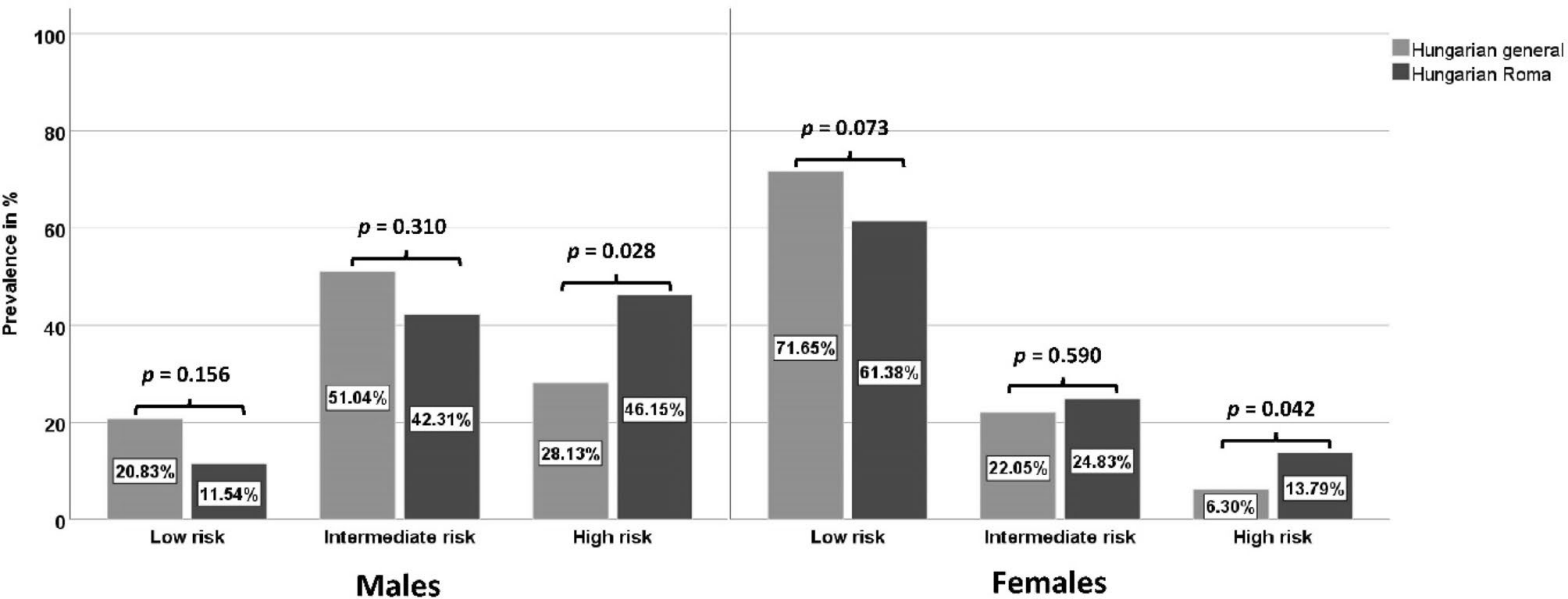
Distribution of SCORE risk categories in the 40–64-year-old Hungarian general and Hungarian Roma populations.

The relative frequency of risk categories in the studied male populations non-significantly differed (p = 0.068). The representation of the high-risk group was more than 1.5 times higher (HG: 28.13% vs. HR: 46.15%, p = 0.028) in the Hungarian Roma male population than in the general one. No significant difference was detected in the distribution profile of risk frequencies between the female populations (p = 0.082), however, there are significantly more people belonging to the high-risk group among Roma females than among Hungarians (HG: 6.30% vs. HR: 13.79%, p = 0.042), for more details see Fig. 3.

### Comparison of estimated 10-year risk based on ACC/AHA Pooled Cohort Equations (PCE) and Revised Pooled Cohort Equations (RPCE) between Hungarian general and Roma populations

The average 10-year risk based on PCE and RPCE show a significant difference between the Hungarian general and Roma male populations (PCE: HG: 7.81% vs. HR: 10.39%, p = 0.012; RPCE: HG: 6.64% vs. HR: 9.48%, p = 0.020). Estimated cardiovascular risk also showed significant difference in case of the study female populations (PCE: HG: 3.55% vs. 5.59%, p < 0.001; RPCE: 2.43% vs. 4.16%, p = 0.004). See more details in Table 6.

**Table 6:** Results obtained by using ACC/AHA pooled cohort equations (PCE) and revised pooled cohort equations (RPCE) in the 40–64-year-old Hungarian general and Hungarian Roma populations.

	Males			Females		
	Hungarian general (n = 96)	Hungarian Roma (n = 52)	p-value	Hungarian general (n = 127)	Hungarian Roma (n = 145)	p-value
	Mean (95% CI)			Mean (95% CI)		
PCE	**7.81 (6.55**–**9.07)**	**10.39 (8.38**–**12.41)**	**0.012**	**3.55 (3.01**–**4.09)**	**5.59 (4.75**–**6.43)**	**< 0.001**
RPCE	**6.64 (5.61**–**7.68)**	**9.48 (7.34**–**11.63)**	**0.020**	**2.43 (2.02**–**2.84)**	**4.16 (3.31**–**5.00)**	**0.004**

Significant differences in mean or prevalence rates are highlighted in bold.

**Figure 4:**
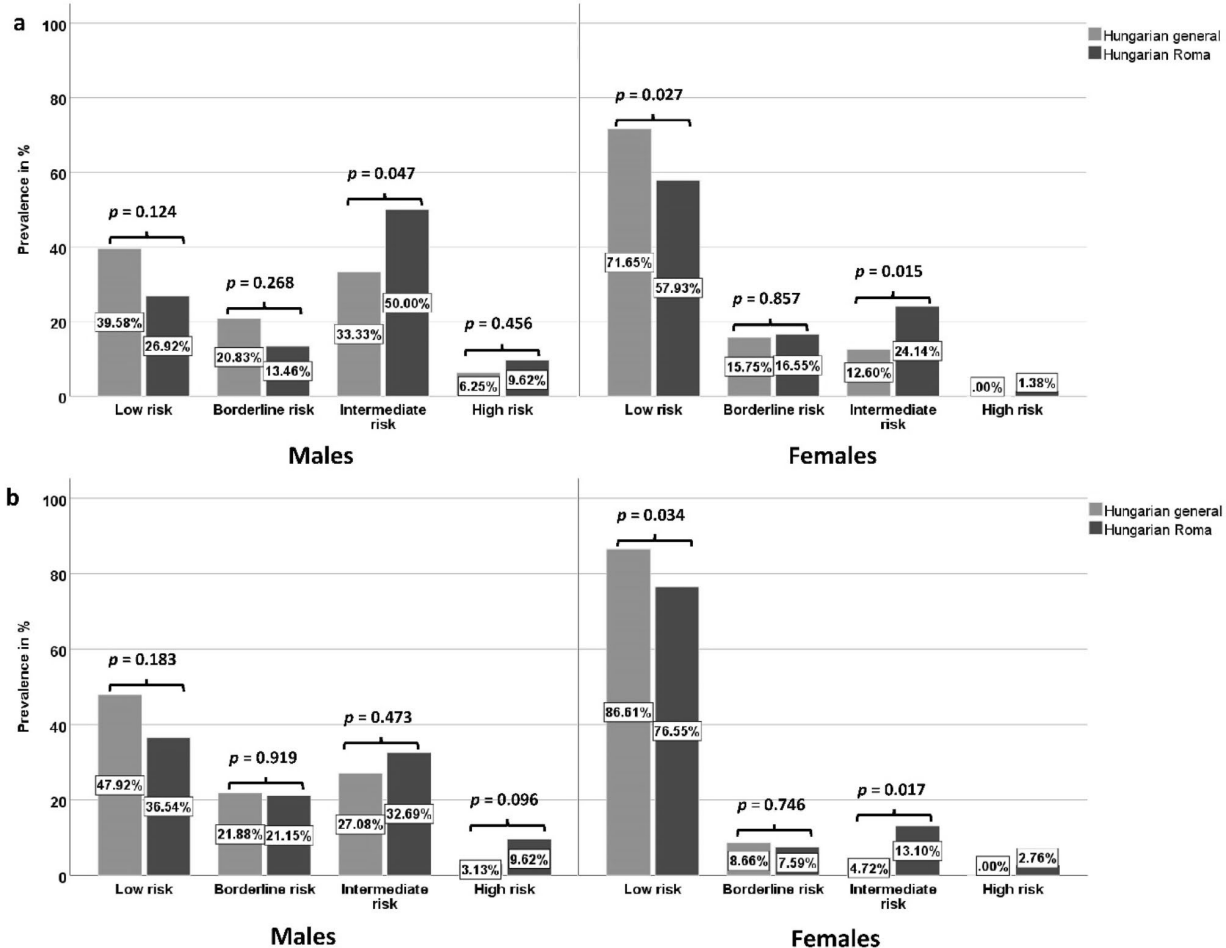
Distribution of subjects by risk categories defined by ACC/AHA pooled cohort equations (PCE-a) and revised pooled cohort equations (RPCE-b) in the 40–64-year-old Hungarian general and Hungarian Roma populations.

The relative frequency of low-, intermediate-, and high-risk subjects in the studied male populations did not differ significantly in either score calculations (PCE: p = 0.142 or RPCE: p = 0.262). The prevalence of the high-risk group was 1.5 times higher in case of PCE (HG: 6.25% vs. HR: 9.62%, p = 0.456) and three times in case of the RPCE (HG: 3.13 vs HR: 9.62, p = 0.096) in the Hungarian Roma male population than in the general one. There were significant differences in the relative frequency of risk groups between the female populations in the case of both versions of the Pooled Cohort Equations (PCE: p = 0.036 and RPCE: p = 0.022). The prevalence of the intermediate-risk group was significantly 2 times higher in case of PCE (HG: 12.60% vs. HR: 24.14%, p = 0.015) and 2.8 times in case of the RPCE (HG: 4.72% vs HR: 13.10%, p = 0.017) in the Hungarian Roma male population than in the general one. A high-risk individual occurred only in the Hungarian Roma male population (PCE: 1.38%; RPCE: 2.76%). See more details in Fig. 4.

## Discussion

The incidence and prevalence of cardiovascular diseases, as well as the mortality caused by them in the Hungarian population, are extremely high compared to other EU member states^2^. It is reasonable to suppose that the unfavourable morbidity and mortality figures strongly related to the fact that about 8–10% of the Hungarian population belongs to the Roma ethnic group. It is well documented that the prevalence of traditional cardiovascular risk factors is higher among the Roma than in the general population. Although previous studies have already estimated the frequency of cardiovascular risk factors among the Roma, no study has yet been conducted with the aim of examining the combined effects of these risk factors by using any of the CVD risk assessment models.

At present, there are multiple risk stratification algorithms in use to estimate the cardiovascular risk at both an individual and a population level. Our main goal was to perform a cardiovascular risk assessment in the Hungarian general and Roma populations and compare the results obtained.

To achieve this goal, the 10-year risk of developing coronary artery disease (based on Framingham Risk Scores), fatal atherosclerotic events (based on Systematic COronary Risk Evaluation), fatal coronary heart disease, nonfatal myocardial infarction, and fatal and non-fatal stroke (based on Pooled Cohort Equations) were estimated in both study populations.

Approximately half of all deaths in Hungary can be attributed to behavioural risk factors, such as alcohol consumption, smoking, poor diet, and low physical activity^59^. The proportion of these factors is well above the EU average of 39%. In 2014, the daily smoking rate was 25%, making it the third highest in the EU. Approximately every fifth death was attributable to tobacco consumption (direct or second-hand smoking). In 2017, every fifth adult was obese in Hungary, making adult obesity one of the highest in the EU. The proportion of the obese in the population has been increasing over the past decade. Overweight and obesity among children is also a growing problem, with nearly one in five 15-year-olds being overweight or obese in 2013 and 2014^60^. In 2017, dietary risks (high sugar and salt intake, low fruit and vegetable consumption) were also responsible for nearly 28% (~ 34 000) of total mortality, which is 10% higher than the EU average^59^.

Our study confirms the fact that the prevalence of cardiovascular risk factors are higher among Roma. In the Roma population, we observed a significantly higher prevalence of smoking regardless of sex or age which is partly attributable to the fact that smoking is a part of the traditional Roma lifestyle^61,62^. The proportion of Roma females being under antidiabetic therapy (30–64-year old: HG: 8.59% vs. HR: 16.33% p = 0.029; 40–64-year old: HG: 11.02% vs. HR: 18.62%, p = 0.081) was higher furthermore the HDL-C level was significantly lower (30–64-year old: HG: 1.47 vs. HR: 1.29, p < 0.001; 40–64-year old: HG: 1.48 vs. HR: 1.31, p < 0.001) in both age groups. According to our previous research, the higher prevalence of low HDL-C levels among Roma is clearly due to genetic causes^31–33^, while the factors behind diabetes are due to ethnicity related ones^34^. Our results that smoking and low HDL-C levels are more common among Roma than in the general population are in harmony with those previously published on the topic^13,15,16,20,23,25^.

In our study, it was found that the 10-year cardiovascular risk of the Roma population independently from the gender is significantly higher compared to that of the Hungarian general one regardless of the risk estimator model used.

Classifying the population as low-, intermediate-, or high-risk groups from a cardiovascular point of view is an important strategy to identify individuals susceptible to serious cardiovascular events. It is also a useful way of identifying people whose health may be improved by preventive measures. Apart from being time- and cost-effective, this minor intervention can further reduce the chances of developing a cardiovascular disease^63,64^, therefore, decreases cardiovascular mortality. Hence, we can shift our focus on primary prevention methods (e.g.: reduction of smoking, taking aspirin, antihypertensive and cholesterol-lowering preventive medication in the indicated groups, and/or reducing body weight) instead of treating CVD^65,66^.

In the case of both sexes, it can be said that the share of individuals belonging to the high-risk group was significantly higher in the case of the Roma population compared to the Hungarian general one. In the case of the Roma population, the proportion of the high-risk group was on average 1.5–3 times higher than that of the Hungarian general one.

Our current study has its strengths and limitations. On the one hand, the accurate identification of ethnicity is a common challenge for studies like ours. Due to the criteria of sample selection used in our present study (individuals were excluded during the age-matching process and if they were diagnosed with any form of CVD), the sample population cannot be interpreted as a representative sample for the whole Hungarian general or Roma population. Given that the Hungarian general population may also include Roma individuals, the difference between the two groups may be underestimated. Risk assessment models used in the study include only traditional cardiovascular risk factors, in other words, age, gender, smoking and diabetic status, blood pressure, and cholesterol levels. This represents a major limitation when applying these equations to genetically susceptible people. The risk assessment models were validated/tested neither for the Hungarian general nor for the Roma population, so the reliability of our results might be questioned.

On the other hand, the study has several strengths. This is the first study to compare the cardiovascular risk burden of the Hungarian general and Roma populations. In our study samples were collected from the same geographical area, hence significant confounding factors on CVD risk, such as access to health care or specific environmental exposures can be excluded.

It is clear from our results that, regardless of the risk calculation method used, higher cardiovascular risk can be detected among the Hungarian Roma compared to the general population. The Roma living in Hungary should be considered highly endangered also from a cardiovascular point of view, and this should be taken into account especially when developing targeted cardiovascular interventions. The extremely high overall cardiovascular risk among Roma men should also be highlighted, which is in harmony with the high prevalence of premature mortality caused by CVDs among Roma^67,68^.

## Data Availability

The datasets generated and/or analysed during the current study are not publicly available due to privacy/ethical restrictions but are available from the corresponding author on reasonable request.
